# Immediate Deep Inferior Epigastric Perforator Flap Reconstruction of a Giant Phyllodes Tumor

**DOI:** 10.7759/cureus.31685

**Published:** 2022-11-19

**Authors:** Brandon Larson, Andrew Francis, Meghan Brown, Victoria Van Fossen, Derek Cody

**Affiliations:** 1 General Surgery, Summa Health, Akron, USA; 2 Plastic and Reconstructive Surgery, Summa Health, Akron, USA; 3 Breast Surgery, Summa Health, Akron, USA; 4 Plastic and Reconstructive Surgery, Crystal Clinic Orthopaedic Center, Akron, USA

**Keywords:** breast benign and malignant surgery/ conditions, giant phyllodes tumour, deep inferior epigastric perforator flap, breast cancer, breast oncology, diep flap, breast reconstruction, giant phyllodes

## Abstract

We present a case of a giant phyllodes tumor (PT) requiring simple mastectomy with en bloc pectoralis major resection and immediate deep inferior epigastric perforator (DIEP) flap reconstruction. This patient presented with a four-year history of an enlarging breast mass with ultrasound-guided biopsy results consistent with atypical fibroepithelial proliferation that was highly concerning for a borderline phyllodes tumor. In this large, rare breast tumor that required en bloc pectoralis major resection, we describe the novel use of an immediate single pedicled DIEP flap for the resulting chest wall defect. The patient’s postoperative course was uncomplicated, and she reported satisfactory cosmetic and functional outcomes at her initial postoperative follow-up visits. Our findings support the use of simple mastectomy with en bloc resection and immediate single-pedicled DIEP flap for the definitive treatment of giant phyllodes tumors. Our experience shows this is a safe and effective technique for achieving adequate oncologic resection while maintaining postoperative function and cosmesis, which are essential for patient quality of life.

## Introduction

Phyllodes tumors (PTs) are fibroepithelial neoplasms that account for less than 1.0 % of all primary breast tumors [1]. The standard of care is wide local excision with 1 cm margins and careful consideration of adjuvant therapy to prevent local recurrence (LR). The goal of oncologic resection is balanced with an acceptable cosmetic outcome, which can be achieved for tumors up to 5 cm. However, approximately 20% of PTs are greater than 10 cm and are defined as giant phyllodes tumors [2]. Giant PTs are often not amenable to breast-conserving approaches and require a mastectomy, which results in significant soft tissue defects that may require reconstruction 

We present a case of a giant phyllodes tumor requiring simple mastectomy and excision of the pectoralis major muscle with immediate deep inferior epigastric artery perforator (DIEP) flap reconstruction.

## Case presentation

**Figure 1:**
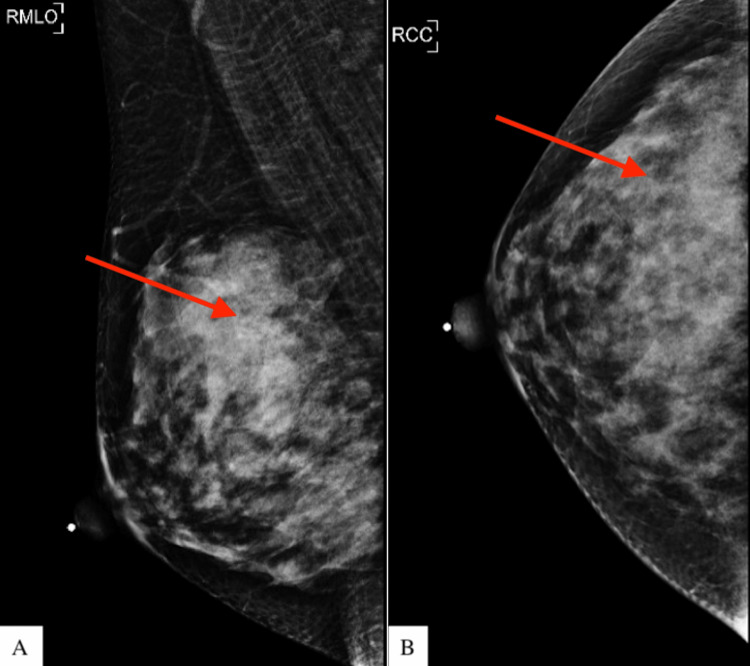
Screening mammogram four years preoperatively that showed an obscured mass in the upper outer quadrant of the right breast. A) right mediolateral oblique view; B) right craniocaudal view RMLO: right mediolateral oblique; RCC: right craniocaudal

A 51-year-old female was referred to the outpatient breast clinic for an enlarging and painful right breast mass. Four years ago, she underwent a screening mammogram that showed an obscured mass in the upper outer quadrant of the right breast (Figure 1).

**Figure 2:**
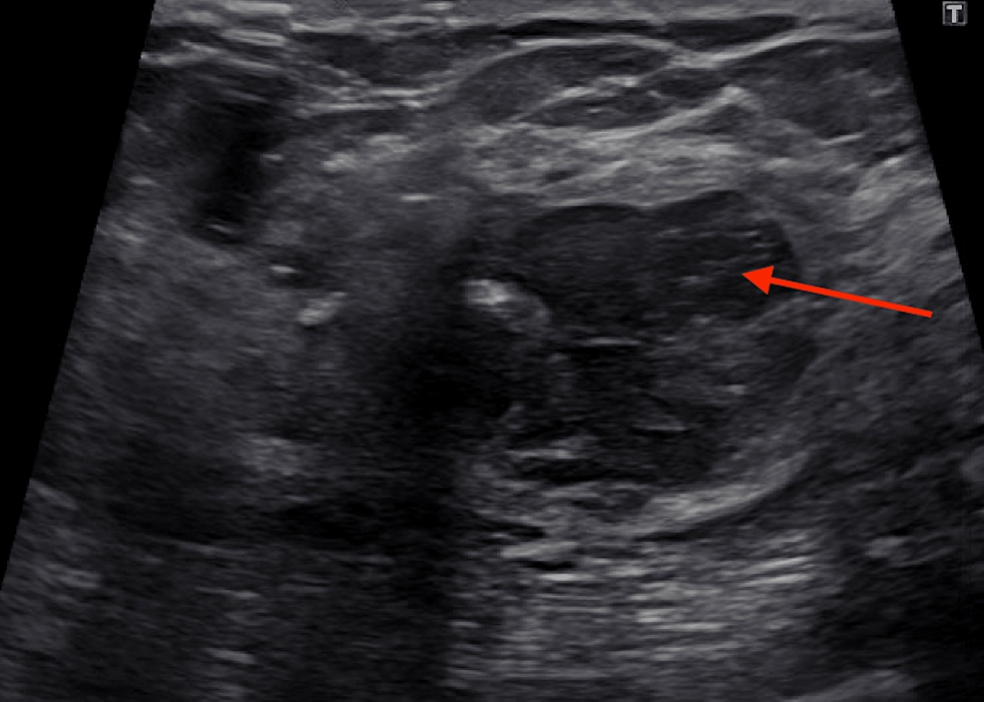
A 2.8 cm hypoechoic mass at the 10:00 position, 5 cm from the nipple, was discovered on a four-year preoperative right breast ultrasound. The mass was subsequently biopsied.

A follow-up right breast ultrasound demonstrated a 2.8 cm hypoechoic mass at the 10:00 position, 5 cm from the nipple (Figure 2).

**Figure 3:**
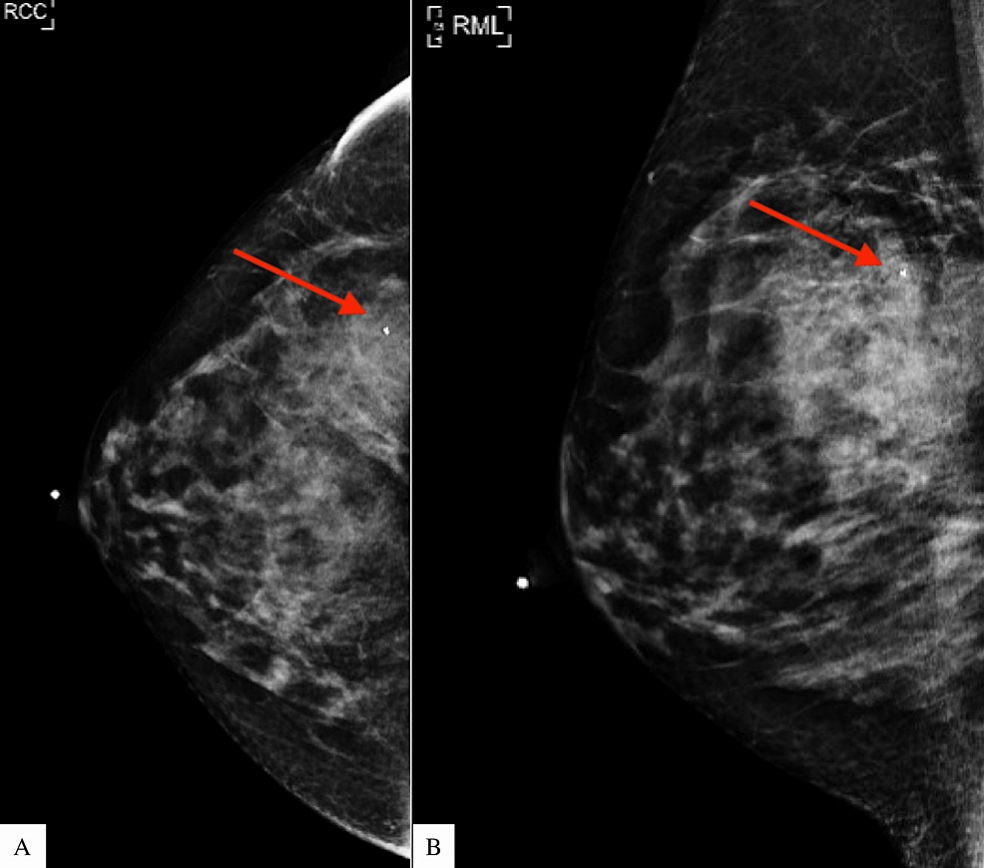
Three and a half years of preoperative diagnostic mammography demonstrated the lesion had increased to 3.6 x 2.2 x 3.0 cm from 2.8 x 2.3 x 1.9 cm. A) right craniocaudal view; B) right mediolateral view RCC: right craniocaudal; RML: right mediolateral

An ultrasound-guided biopsy was performed, and histopathology showed a fibroepithelial proliferative lesion consistent with a proliferating or juvenile fibroadenoma. The patient was recommended to have short-interval follow-up imaging. A six-month diagnostic mammogram demonstrated the lesion had increased to 3.6 x 2.2 x 3.0 cm from 2.8 x 2.3 x 1.9 cm (Figure 3).

**Figure 4:**
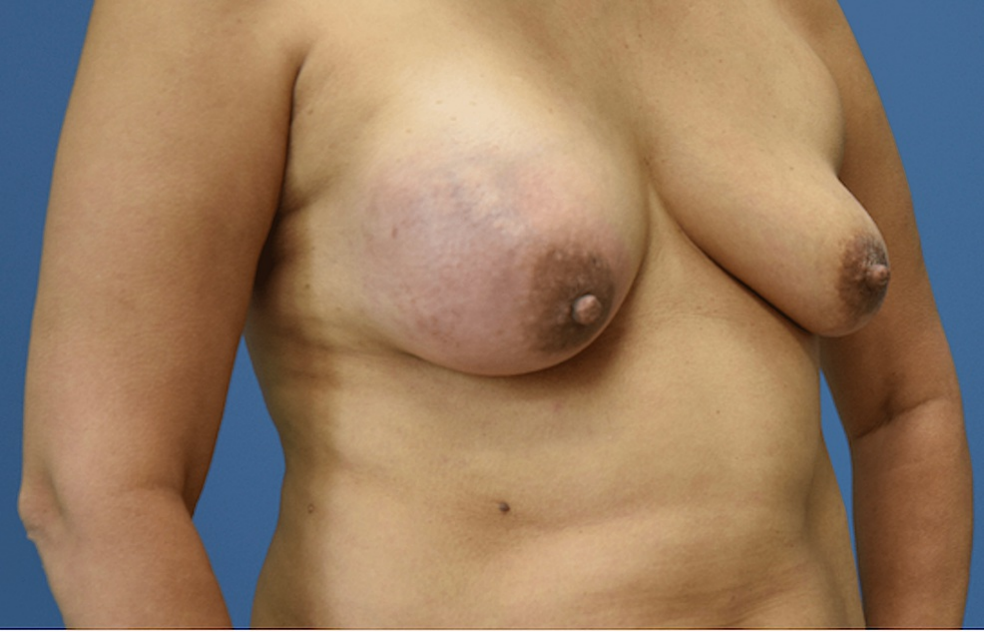
Preoperative view of a giant phyllodes tumor in the right breast

Surgical excision was recommended at that time; however, she had not followed up for any breast imaging or surgical excision. Since then, she has noted the continued growth of the mass over the past three years, with a rapid increase in size over the last two to three months. Her personal and family history was unremarkable. Laboratory analysis was notable for anemia at a baseline of 8.8 g/dL. On examination, her right breast was markedly enlarged and tender with a firm, palpable mass occupying the breast, measuring approximately 15 cm. The overlying skin was shiny without significant ulceration, cellulitis, or abscesses (Figure 4). There was no evidence of clinically positive lymph nodes or nipple discharge.

**Figure 5:**
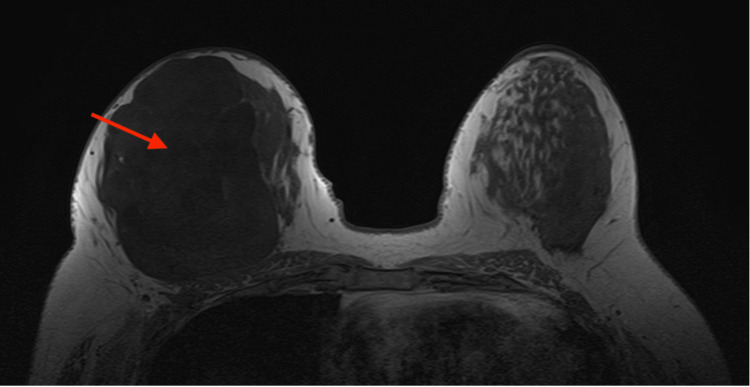
Preoperative breast MRI revealing a right-sided 10.9 x 9.4 x 11.7 cm heterogenous T2-enhancing lobulated mass that occupies the entire right breast.

A subsequent breast MRI revealed a right-sided 10.9 x 9.4 x 11.7 cm heterogenous T2-enhancing lobulated mass that occupied the entire right breast (Figure 5). A metastatic workup with computed tomography (CT) of the chest did not reveal any distant metastasis. A bilateral ultrasound revealed a contralateral 1.2 x 0.5 x 0.9 cm hypoechoic mass at the 11:00 position, 4 cm from the nipple. As such, she underwent a bilateral ultrasound-guided biopsy. Histopathology was notable for atypical fibroepithelial proliferation that was highly concerning for borderline PT of the right breast and a fibroadenoma of the left breast.

**Figure 6:**
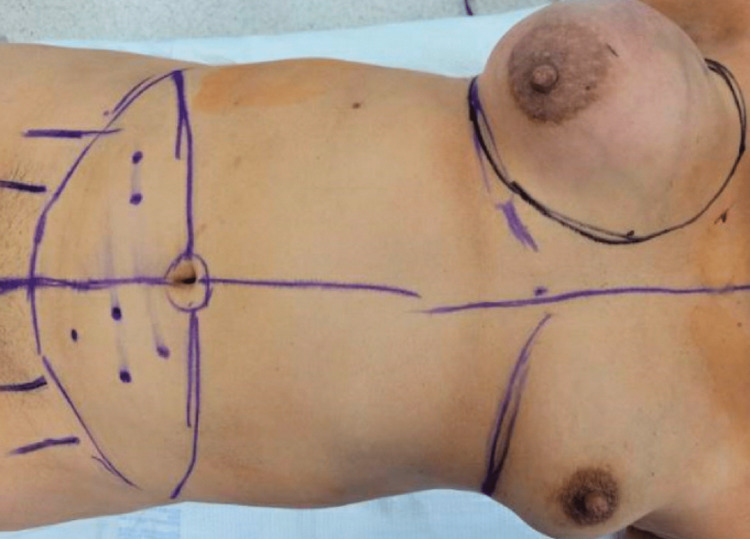
Preoperative markings for right simple mastectomy and DIEP flap reconstruction DIEP - Deep Inferior Epigastric Perforator

**Figure 7:**
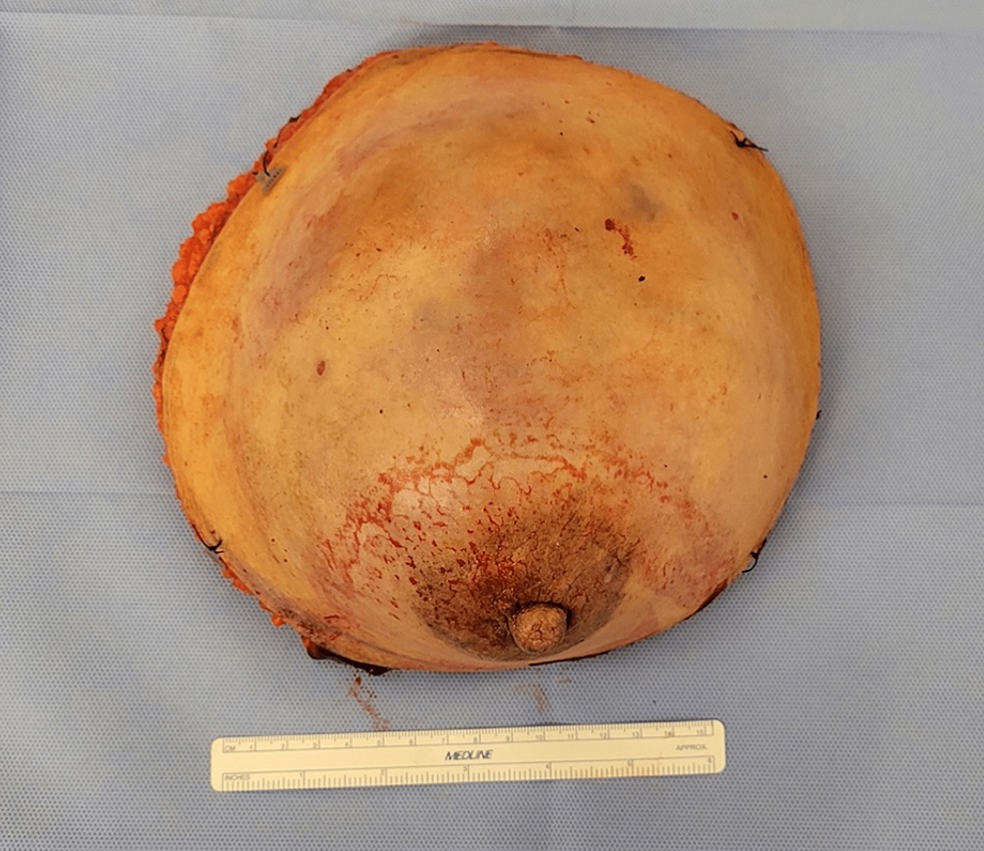
Surgical specimen weighing 1,954 g with dimensions of 13.5 x 10 x 12 cm

**Figure 8:**
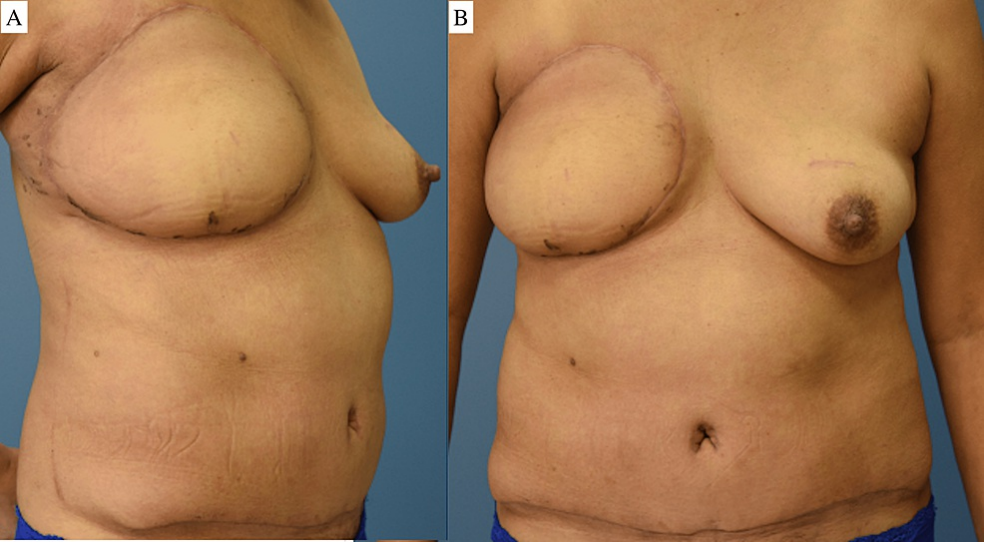
Two weeks postoperatively from a simple mastectomy with en bloc pectoralis major resection and immediate DIEP flap reconstruction. A) right anterior oblique view; B) anterior view DIEP - deep inferior epigastric perforator

The case was discussed preoperatively at a multidisciplinary tumor board, and based on the large tumor size, proximity to the nipple-areolar complex on MRI, and patient preference of surgical options, a simple right mastectomy with immediate DIEP flap reconstruction and left excisional biopsy was performed (Figure 6). The posterior aspect of the mass was close to the pectoralis muscle; therefore, en bloc resection of the pectoralis muscle was taken down to the chest wall. The specimen weighed 1,954 g, and its dimensions were 13.5 x 10 x 12 cm (Figure 7). After resection, a free DIEP flap was dissected out, and perfusion was evaluated intraoperatively with indocyanine green and the Spy Elite® fluorescence imaging system (Stryker®, Kalamazoo, MI). A single perforator was anastomosed to the internal mammary artery at the third intercostal space. The deep inferior epigastric vena comitantes and superficial inferior epigastric vein were anastomosed with a 2.5-mm coupler to the lateral and medial internal mammary veins, respectively. The patient recovered well postoperatively, and the flaps remained healthy and viable throughout her hospital stay. She was discharged on postoperative day three. At the two-week postoperative follow-up appointment, it was noted that the patient had recovered well without any concerns (Figure 8).

**Figure 9:**
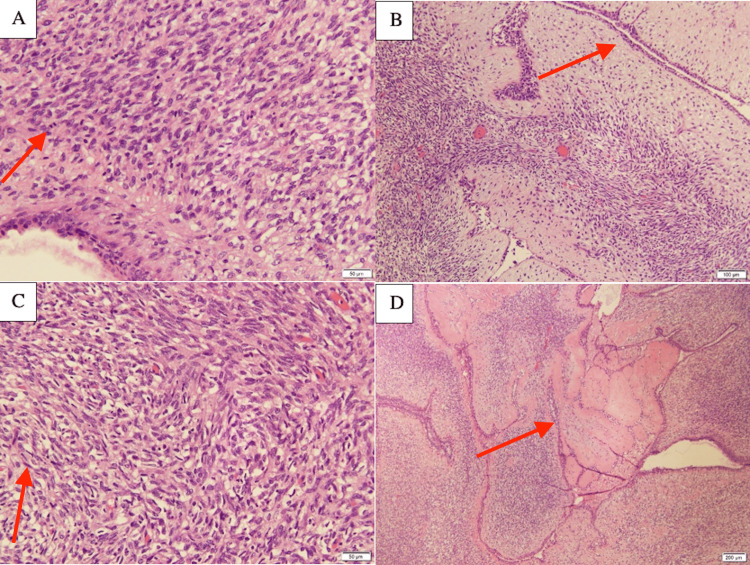
(A) 20X magnification reveals a cellular stromal component of the lesion. Stromal cells are spindly in appearance with pleomorphism and hyperchromasia. Mitotic figures are also up. B) A benign epithelial component with adjacent fascicles of cellular stroma at 10X magnification. C) 20X magnification of the stroma, which is composed of spindle cells with nuclear pleomorphism and hyperchromasia. D) A 4X magnification of the lesion reveals an intracanalicular growth pattern of densely cellular stroma pushing the epithelial component, resulting in the typical leaf-like architecture seen in phyllodes tumors.

The left breast excisional biopsy found atypical ductal hyperplasia with microcalcifications and a complex fibroadenoma. The final pathology of the right mastectomy revealed malignant PT with marked ulceration (Figure 9). Margins were negative, with the closest posterior margin at 0.8 cm. Five nodes were removed, including one in the internal mammary node that was negative. Her pathologic stage was IIIB (pT3 N0 M0). She was referred to medical oncology, and upon review of the literature and discussion with the patient, she received adjuvant radiotherapy to the right chest wall.

## Discussion

Phyllodes tumors are uncommon breast neoplasms that Johannes Muller first described in 1838; he initially named them cystosarcoma phyllodes due to their leaf-like stromal projections [3]. The diagnosis and grading of PT are based on recommendations from the World Health Organization (WHO) classification, which separates them based on grade: low grade (benign), intermediate grade (borderline), and high grade (malignant) [4]. Phyllodes tumors have a high tendency for local recurrence (LR) despite surgical resection. Local recurrence typically ranges from 10%-40% and, on average, occurs 18.8 months post-resection. The risk of recurrence is multifactorial and is associated with malignant grade, necrosis, the presence of stomal overgrowth, tumor size, and marked or moderate aplasia. There remains debate on the margin status and local recurrence, as some propose it is dependent on tumor grade [5]. However, current National Comprehensive Cancer Network (NCCN) guidelines recommend wide local excision with 1 cm margins to prevent a recurrence. Further, there is significant heterogeneity with the use of adjuvant radiotherapy, and it should be patient-specific. Currently, no prospective randomized data supports radiotherapy, and the NCCN recommends consideration only where significant morbidity may occur with LR [6].

Prior literature has demonstrated that giant PTs have a higher rate of malignancy compared to smaller tumors, at 42.5% and 21%, respectively. Likewise, the recurrence rate in giant tumors compared to smaller tumors is also increased, at 41% vs. 29%, respectively [7]. Achieving negative 1 cm margins often creates a significant soft tissue defect, making wound closure technically and cosmetically challenging [6].

Few case reports exist regarding autologous reconstruction for the large resultant soft tissue defect following resection of giant phyllodes tumors. Tsuruta et al. describe a similar single-pedicled DIEP-free flap; however, their case differs from ours in that there was not an en bloc resection of the pectoralis major [8]. Fang et al. described the use of a bi-pedicled DIEP-free flap for reconstruction compared to our single-pedicled free flap [9]. DIEP flap reconstruction is the preferred autologous option in patients with sufficient excess abdominal soft tissue, as it provides a large skin paddle and spares the muscles, unlike the transverse rectus abdominis flap and latissimus dorsi flap.

Limitations of the existing literature for autologous reconstruction following resection of giant PT include a direct comparison between various free flap options, the anecdotal nature of case reports, and small sample sizes. There is a need for prospective trials utilizing larger sample sizes to compare recurrence and complications. However, this is logistically challenging given the nature of such a rare disease.

This case demonstrates that the resection of a giant phyllodes tumor with immediate single-pedicled DIEP flap reconstruction can be performed with satisfactory oncologic, functional, and cosmetic results.

## Conclusions

The giant phyllodes tumor is a rare breast neoplasm and is often challenging to manage from a resection and reconstruction standpoint. Previous case reports describe a simple mastectomy with DIEP flap reconstruction. We present the results of a mastectomy with en bloc pectoralis primary resection and a single pedicled DIEP free flap reconstruction.

Our experience shows this is a safe and effective technique for achieving adequate oncologic resection while maintaining postoperative function and cosmesis, which are essential for patient quality of life. We propose this technique for the definitive management of giant phyllodes tumors of the breast.
